# Critical evaluation of current diagnostic systems

**DOI:** 10.4103/0019-5545.43621

**Published:** 2008

**Authors:** Claudio E. M. Banzato

**Affiliations:** Department of Psychiatry, Medical School, University of Campinas (Unicamp), Campinas, Brazil

In a way, the current state of affairs would surpass Stengel's best expectations in the late fifties as internationally adopted classifications (ICD/DSM) have indeed become psychiatry's ‘common language’. However, by ignoring Stengel's proviso that read ‘it [an international classification] must be a servant of international communication rather than its master’, we seemingly ended up with an ICD/DSM supremacy.[1] Internationally adopted classifications have not only become lately the rulers of communication, but also have shaped psychiatric clinical practice, professional education and research programs to a great extent. As high hopes were placed in such a move for a myriad of legitimate reasons, one should inquire about its actual payoff.

Perhaps a major disappointment to date with current classificatory systems is the fact that most of their diagnostic categories simply failed to be validated, either by the discovery of specific etiologies (or pathophysiologic pathways)[2] or by a robust convergence of independent indicators.[3] As it turned out, validators, contrary to early beliefs, do not appear to be monolithic.[4] Accordingly, the causation of mental disorders is most likely a multifactorial process, which means we have to account for many factors from different sorts (each one itself with somewhat weak causal influence) that interact within very complex etiologic networks.[3,5]

Regarding psychiatric epidemiology, there were obvious gains in the last decades.[6] Data on the prevalence of mental disorders worldwide have become available. Though comparison across countries and regions may not be fully granted due to issues related to the still uncertain cultural sensitivity (and meaningfulness) of the current diagnostic tools, the estimate rates obtained so far have played an important role in the development of mental health policies. In addition, sophisticated epidemiological methods have been conceived to inform the revision of current diagnostic systems by testing the assumption of the existence of discrete entities and by helping us to select optimal cut-points for defining diagnostic criteria.[7] It should be noticed that although most criteria sets in ICD-10 and DSM-IV are similar, very few actually coincide. Sorting out meaningful differences from the insignificant ones is a necessary step for the empirical comparative testing of the key options made by each system.[4]

The consequences of the current diagnostic systems on clinical practice and on teaching and training psychiatry are possibly harder to assess. On the up side, it is usually agreed that they have contributed to strengthen the medical identity of psychiatry and to demystify psychiatric diagnosis to some extent, which may have led to a reduction in the stigma associated with mental disorders.[6] With regard to adverse outcomes, several points have also been made, among them the conflict between clinical and research goals[8] and the fact that they are often used in a *cookbook* fashion. Thus, it would be fair to try to separate which untoward effects are due to the intrinsic problems of such systems from those secondary to their simple misuse, even though such sifting may eventually prove unwarranted. Perhaps it would be interesting to make a further distinction (that cuts across the former one) between problems related to the classificatory scheme (taxonomy) and problems related to the diagnostic guidance provided (diagnostic model at their core).

The inflation of the comorbidity rates has been probably the most controversial consequence of present classifications. But it has now become clear that such inflation occurred largely at the expenses of artifactual co-morbidity, a by-product of the (predominantly, because it does not apply across all categories) splitting strategy adopted in DSM and ICD.[4] It should be added that exclusion criteria were meant to counterbalance this trend, but because they were stipulated (not based on empirical grounds); the nosological hierarchy built-in into the system was not warranted. As a matter of fact, the presence of exclusion criteria makes the validity of diagnostic categories mutually interdependent.[9] To the extent that they have been dropped in the last DSM revisions, artifactual co-morbidity has become even more pervasive.[10]

These rule-based classifications with explicit set of criteria did enhance reliability of communication, but one may remain a bit skeptical about the actual use of diagnostic criteria. The apprehension of psychopathological phenomena involves a good deal of clinical interpretation and judgment. Psychiatric symptoms are typically unspecific and they are far from being self-evident building blocks of higher-order syndromes; not to mention their conspicuous lack of temporal stability. In psychiatry, hyponarrativity does not necessarily ensure objectivity. Furthermore, there is no guarantee that diagnostic criteria are used in the same fashion. Indeed, it is often said that clinicians divide concerning the use of diagnostic criteria: those who are still in training are more likely to count symptoms, while the experienced ones tend to rely more on prototypes.

Both ICD and DSM have multiaxial schemes. The adoption of such approach was perceived as a positive development in terms of comprehensive diagnostic assessment. Actually, the DSM multiaxial system fostered a renewal in the interest for personality disorder studies and it contributed to an increase in the registration of general medical conditions in psychiatric patients' clinical charts as well.[11] The ICD-10 multiaxial system[12] established a very interesting connection with other instruments also developed by WHO (taken together they comprise a family of classifications): axis II borrows from the Disability Assessment Scale (WHO DAS-S); axis III takes advantage of the ICD-10 Z-codes.

Much of the debate on classification of course gravitates around the demarcation of the boundaries between different disorders. These discussions take place within either a naturalistic framework (pursue of the correct classification) or a pragmatic one (search for the best way to classify certain given purposes). Whilst such complicated issue concerns mostly nosologists, it attracts a lot of attention from clinicians at large as they (legitimately) seem to entertain high hopes that better classification will make their everyday clinical practice somewhat easier. In a way, overemphasis on classification of disorders may be detrimental to the whole process of diagnostic assessment, where what is really at stake is the full apprehension of the clinical picture presented by the patient. In that regard, it should be mentioned the comprehensive diagnostic model put forward by WPA in its International Guidelines for Diagnostic Assessment (IGDA), which building on the ICD scheme incorporates a fourth axis, quality of life, plus an idiographic (narrative) formulation.[13,14]

A neglected aspect in the nosographic literature is the constraints that diagnostic categories and classificatory schemes may impose (either intentionally or inadvertently) on the clinical encounter. People act under descriptions, that is, evolving human experience is shaped within a given frame of reference (cultural background). Therefore, the framework used for such descriptions matters a great deal.[15] Psychiatric classification and nomenclature, built and first adopted by mental health professionals, gradually become ingrained in the culture. Eventually, not only lay people start using psychiatric categories and terms of art, but also having their own subjective experience reframed accordingly. Diagnostic labels are presently being used by the patients to name their complaints. On the side of the clinicians, reification has also hardly been resisted. So, what kind of psychiatric clinics can actually take place within such a context?

A correlated question pops up: how do these descriptive classificatory tools affect conceptualization and teaching of psychopathology? What about the lack of consideration for the developmental dimension, the changing psychopathology over the life span?[16] Have we sacrificed the subtleties and complexities of morbid subjective experience at the shrine of reliability?[17] According to Jaspers, the subject-matter of psychopathology is the whole man in his infirmity.

Another important domain that has received scholar attention lately is the implicit values and assumptions (aesthetic, ethical, pragmatic, epistemic and ontological) of classificatory systems. Major efforts have been made to unpack and reveal them.[18] In addition, the roles of values (along with facts) in diagnosis have been underscored by philosophers of psychiatry.[19,20] The same authors have also presented actual contributions to psychiatry from philosophy in the following areas: patient-centered practice, new models of service delivery, neuroscience research, psychiatric education, and the organization of psychiatry as an international science-led discipline focused on patient care.[21]

In sum, it has become clearer that building a more valid and useful psychiatric nosology seemingly depends on many different kinds of factors. Thus, the plea for the combination of sophisticated conceptual framework, methodological and explanatory pluralism and sound scientific empirical evidence.[22,23]

Two questions before finalizing this sketch. The first is germane to the exam of our current diagnostic systems: to whom are they actually serving, who are their stakeholders and what role should they play in the development of such tools?[24] The second one is more general and concerns the purposes of psychiatry: what are our working (hidden) concepts of ‘man’ and ‘fulfilled life’ (eudaimonia)?[18] Everything else in psychiatry (diagnostic and classificatory tools included) hinges on the way we will address them.

It is crucial then to focus on the purposes of diagnosis and on the extent to which the different available diagnostic models serve such goals. As medicine and psychiatry are modificatory activities, patients should be the ultimate stakeholders of the diagnostic systems. Thus, the suggestion that the development of comprehensive schemes, presumably better suited to inform clinical care,[13,25] should become a priority for psychiatry.
